# {*S*-Benzyl 3-[(6-methyl­pyridin-2-yl)­methyl­idene]dithio­carbazato}nickel(II) monohydrate

**DOI:** 10.1107/S1600536812006952

**Published:** 2012-02-24

**Authors:** Siti Aminah Omar, Thahira B. S. A. Ravoof, Mohamed Ibrahim Mohamed Tahir, Karen A. Crouse

**Affiliations:** a Department of Chemistry, Faculty of Science, Universiti Putra Malaysia, 43400 UPM Serdang, Selangor, Malaysia

## Abstract

The structure of the title compound, [Ni(C_15_H_14_N_3_S_2_)_2_]·H_2_O, has one mol­ecule in the asymmetric unit, along with a solvent water mol­ecule. The two different Schiff base moieties coordinate to the central Ni^II^ ion as tridentate *N*,*N*′,*S*-chelating ligands, creating a six-coordinate distorted octa­hedral environment [the smallest angle being 77.43 (7)° and the widest angle being 169.99 (7)°]. The mean planes of the two ligands are nearly orthogonal to each other with an angle of 89.53 (6)°. The packing of the complex is supported by O—H⋯N and O—H⋯S hydrogen bonding between the solvent water mol­ecule and the uncoordinated N and S atoms of neighbouring ligands.

## Related literature

For background on the coordination chemistry of hydrazine carbodithio­ates, see: Ravoof *et al.* (2010 ▶). For the synthesis, see: Ali *et al.* (1997 ▶). For related structures, see: Khoo *et al.* (2005 ▶); Paulus *et al.* (2011 ▶).
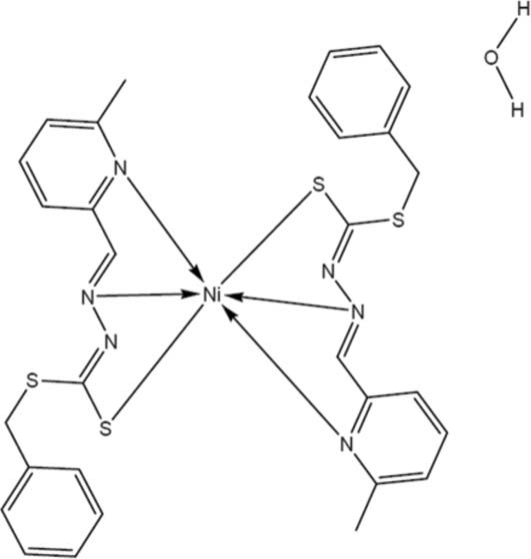


## Experimental

### 

#### Crystal data

[Ni(C_15_H_14_N_3_S_2_)_2_]·H_2_O
*M* *_r_* = 677.58
Monoclinic, 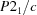

*a* = 16.2165 (5) Å
*b* = 13.2474 (3) Å
*c* = 15.5935 (3) Å
β = 111.404 (3)°
*V* = 3118.86 (14) Å^3^
*Z* = 4
Mo *K*α radiation
μ = 0.93 mm^−1^
*T* = 150 K
0.37 × 0.12 × 0.06 mm

#### Data collection

Oxford Diffraction Gemini CCD diffractometer
Absorption correction: multi-scan (*CrysAlis RED*; Oxford Diffraction, 2002 ▶) *T*_min_ = 0.89, *T*_max_ = 0.95
20706 measured reflections
7223 independent reflections
6011 reflections with *I* > 2σ(*I*)
*R*_int_ = 0.032

#### Refinement

*R*[*F* ^2^ > 2σ(*F* ^2^)] = 0.036
*wR*(*F* ^2^) = 0.087
*S* = 0.97
7199 reflections
379 parameters
H-atom parameters constrained
Δρ_max_ = 0.53 e Å^−3^
Δρ_min_ = −0.74 e Å^−3^

### 

Data collection: *CrysAlis CCD* (Oxford Diffraction, 2002 ▶); cell refinement: *CrysAlis RED* (Oxford Diffraction, 2002 ▶); data reduction: *CrysAlis RED*; program(s) used to solve structure: *SIR92* (Altomare *et al.*, 1994 ▶); program(s) used to refine structure: *CRYSTALS* (Betteridge *et al.*, 2003 ▶); molecular graphics: *CAMERON* (Watkin *et al.*, 1996 ▶); software used to prepare material for publication: *CRYSTALS*.

## Supplementary Material

Crystal structure: contains datablock(s) global, I. DOI: 10.1107/S1600536812006952/wm2591sup1.cif

Structure factors: contains datablock(s) I. DOI: 10.1107/S1600536812006952/wm2591Isup2.hkl

Additional supplementary materials:  crystallographic information; 3D view; checkCIF report

## Figures and Tables

**Table 1:** Selected bond lengths (Å)

N102—Ni1	2.0127 (18)
S106—Ni1	2.4259 (5)
N115—Ni1	2.1787 (16)
N202—Ni1	2.0197 (18)
S205—Ni1	2.4211 (6)
N215—Ni1	2.1784 (17)

**Table 2:** Hydrogen-bond geometry (Å, °)

*D*—H⋯*A*	*D*—H	H⋯*A*	*D*⋯*A*	*D*—H⋯*A*
O301—H3011⋯S205	0.91	2.42	3.323 (3)	173
O301—H3012⋯N203^i^	0.91	2.04	2.919 (4)	162

Symmetry code: (i) 
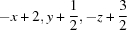
.

**Fig. 1.**
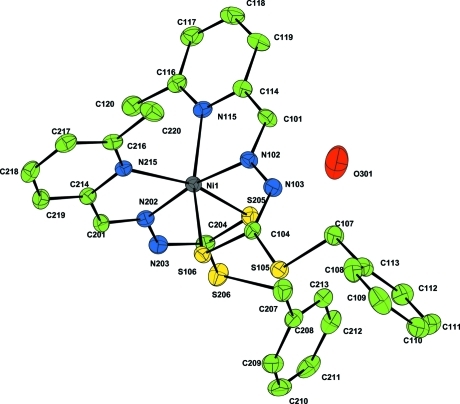
The title compound with displacement ellipsoids drawn at the 50% probability level. H atoms are omitted for clarity.

**Fig. 2.**
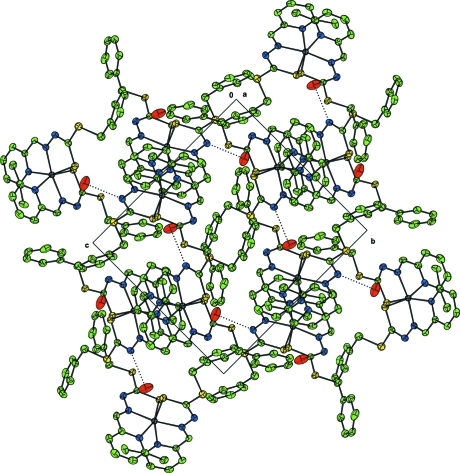
Molecular packing diagram of title compound viewed along the a axis showing the O—H···N hydrogen bonding as dotted lines.

## supplementary crystallographic information

### Comment

The title compound was preferentially formed during the synthesis of the tridentate Schiff base with Ni^II^ saccharinate, by eliminating the saccharinate anion and instead coordinating one metal ion with two tridentate deprotonated Schiff base moieties. Background on the coordination chemistry of hydrazine carbodithioates were given by Ravoof *et al.* (2010). This compound has been previously synthesized by Ali *et al.* (1997), but its crystal structure has not been reported so far.

There is one independent molecule in the asymmetric unit which contains the Ni^II^ ion coordinated to two tridentate Schiff bases *via* the pyridyl nitrogen (N_115, N_215) azomethine nitrogen (N_102, N_202) and thiolate sulfur (S_106, S_205) atoms (Fig. 1). A solvent water molecule in also present in the lattice. The coordination of the metal ion is distorted octahedral with equatorial angles ranging from 77.43 (7)° to 108.62 (7)°. The distortion from the ideal geometry maybe due to the restricted bite angles (80.83 (5) and 81.03 (5) °) of the Schiff base ligands.

The mean planes defined by S106—S105—C104—N103—N102 (largest deviation 0.005 Å) and S206—S205—C204—N203—N202 (largest deviation 0.025 Å) are nearly planar to each other (89.53 (6) °). The torsion angles between the C104—S105—C107—C108 and C204- S206—C207—C208 moieties are -175.11 (18)° and 178.40 (16)° respectively, whereas the torsion angles for the C104—N103—N102—C101 and C204—N203—N202—C201 moieties are -176.7 (2)° and -171.1 (2)° respectively, values close to 180° indicate that the moieties are almost in the same plane.

There is some weak hydrogen bonding (Fig. 2, Table 2) as evidenced by the interaction between the oxygen atom from the independent water molecule with the uncoordinated nitrogen (N_203) and sulfur atoms (S_205) of neighbouring ligands.

For related structures, see: Khoo *et al.* (2005); Paulus *et al.* (2011).

### Experimental

The Schiff base ligand was prepared according to Ali *et al.* (1997). The metal complex of the Schiff base was prepared by adding nickel(II) acetate in hot ethanol (25 ml) to an equimolar quantity of the Schiff base in ethanol (30 ml). The resulting mixture was heated on a water bath until the volume reduced to 30 ml. On standing overnight, the mixture yielded crystals which were filtered off, washed with ethanol and dried in a desiccator over anhydrous silica gel, overnight. The crystals were then dissolved in a solvent mixture of acetonitrile:chloroform in 2:1 mole ratio. To this solution, excess sodium saccharin in water was added (20 ml) in a 1:4 mole ratio. The resulting mixture was heated on a water bath until the volume reduced to 30 ml. On standing overnight inside the fridge, crystals obtained were filtered off, washed with ethanol and dried in desiccator over anhydrous silica gel, overnight. Crystals of the nickel complex suitable for X-ray diffraction analysis were obtained by slow evaporation from a mixture of acetonitrile and THF over a few weeks.

### Refinement

The H atoms were all located in a difference map, but those attached to carbon atoms were repositioned geometrically. The H atoms were initially refined with soft restraints on the bond lengths and angles to regularize their geometry (C—H in the range 0.93–0.98 Å, O—H = 0.82 Å) and *U*_iso_(H) (in the range 1.2–1.5 times *U*_eq_ of the parent atom), after which the positions were refined with riding constraints.

### Crystal data

**Table:**

[Ni(C_15_H_14_N_3_S_2_)_2_]·H_2_O	*F*(000) = 1408
*M**_r_* = 677.58	*D*_x_ = 1.443 Mg m^−^^3^
Monoclinic, *P*2_1_/*c*	Mo *K*α radiation, λ = 0.71073 Å
Hall symbol: -P 2ybc	Cell parameters from 6534 reflections
*a* = 16.2165 (5) Å	θ = 2–29°
*b* = 13.2474 (3) Å	µ = 0.93 mm^−^^1^
*c* = 15.5935 (3) Å	*T* = 150 K
β = 111.404 (3)°	Plate, black
*V* = 3118.86 (14) Å^3^	0.37 × 0.12 × 0.06 mm
*Z* = 4	

### Data collection

**Table:**

Oxford Diffraction Gemini CCD diffractometer	7223 independent reflections
Radiation source: sealed x-ray tube	6011 reflections with *I* > 2σ(*I*)
Graphite monochromator	*R*_int_ = 0.032
combined φ and ω scans	θ_max_ = 29.0°, θ_min_ = 2.2°
Absorption correction: multi-scan (*CrysAlis RED*; Oxford Diffraction, 2002)	*h* = −21→21
*T*_min_ = 0.89, *T*_max_ = 0.95	*k* = −16→17
20706 measured reflections	*l* = −20→21

### Refinement

**Table:**

Refinement on *F*^2^	Primary atom site location: structure-invariant direct methods
Least-squares matrix: full	Hydrogen site location: inferred from neighbouring sites
*R*[*F*^2^ > 2σ(*F*^2^)] = 0.036	H-atom parameters constrained
*wR*(*F*^2^) = 0.087	Method = Modified Sheldrick *w* = 1/[σ^2^(*F*^2^) + (0.04*P*)^2^ + 2.87*P*], where *P* = (max(*F*_o_^2^,0) + 2*F*_c_^2^)/3
*S* = 0.97	(Δ/σ)_max_ = 0.001
7199 reflections	Δρ_max_ = 0.53 e Å^−^^3^
379 parameters	Δρ_min_ = −0.74 e Å^−^^3^
0 restraints	

### Special details

**Table:**

Experimental. The crystal was placed in the cold stream of an Oxford Cryosystems open-flow nitrogen cryostat (Cosier & Glazer, 1986) with a nominal stability of 0.1 K.Cosier, J. & Glazer, A.*M*., 1986. J. Appl. Cryst. 105 107.
Refinement. The number of reflections in the refinement section is 7199 because we used [sin θ/λ]^2^ at least 0.01 to eliminate poor reflections that may be poorly measured in the vicinity of the beam stop. If we removed this condition we will get the 7233 reflections as per data collection with an *R* factor of 3.66% and weighted *R* factor of 8.18%.

### Fractional atomic coordinates and isotropic or equivalent isotropic displacement parameters (Å^2^)

**Table:**

	*x*	*y*	*z*	*U*_iso_*/*U*_eq_	
C101	0.78816 (15)	0.24923 (17)	0.47882 (15)	0.0283	
N102	0.75659 (12)	0.22384 (13)	0.54036 (11)	0.0238	
N103	0.71678 (12)	0.29966 (14)	0.57292 (12)	0.0272	
C104	0.69132 (14)	0.26683 (16)	0.63824 (13)	0.0226	
S105	0.64057 (4)	0.35514 (4)	0.68689 (4)	0.0282	
S106	0.70004 (4)	0.14821 (4)	0.68466 (3)	0.0238	
C107	0.65542 (19)	0.47171 (17)	0.63233 (17)	0.0377	
C108	0.53242 (17)	0.58004 (19)	0.64409 (19)	0.0395	
C109	0.50227 (18)	0.6579 (2)	0.6853 (2)	0.0446	
C110	0.56150 (19)	0.71305 (19)	0.75561 (18)	0.0409	
C111	0.65034 (19)	0.69239 (19)	0.78473 (17)	0.0406	
C112	0.68026 (17)	0.61438 (19)	0.74452 (16)	0.0355	
C113	0.62176 (16)	0.55768 (17)	0.67379 (15)	0.0298	
C114	0.83009 (14)	0.17023 (17)	0.44438 (14)	0.0259	
N115	0.83334 (11)	0.07754 (14)	0.48268 (11)	0.0240	
C116	0.86856 (14)	0.00076 (18)	0.45121 (14)	0.0273	
C117	0.90229 (16)	0.0166 (2)	0.38157 (16)	0.0353	
C118	0.89933 (16)	0.1106 (2)	0.34373 (16)	0.0373	
C119	0.86213 (15)	0.1900 (2)	0.37501 (15)	0.0319	
C120	0.86909 (17)	−0.10138 (18)	0.49139 (17)	0.0350	
C201	0.75877 (14)	−0.11392 (16)	0.65377 (14)	0.0245	
N202	0.81710 (11)	−0.04416 (14)	0.66865 (11)	0.0227	
N203	0.89420 (12)	−0.05579 (14)	0.74432 (12)	0.0262	
C204	0.94462 (14)	0.02487 (17)	0.76176 (14)	0.0248	
S205	0.92301 (4)	0.13799 (4)	0.70535 (4)	0.0270	
S206	1.04398 (4)	0.00134 (5)	0.85547 (4)	0.0333	
C207	1.08635 (16)	0.12604 (19)	0.89890 (16)	0.0355	
C208	1.17032 (15)	0.10646 (17)	0.98114 (14)	0.0280	
C209	1.16698 (17)	0.05990 (19)	1.05927 (16)	0.0348	
C210	1.24391 (18)	0.0352 (2)	1.13128 (15)	0.0391	
C211	1.32515 (17)	0.05774 (19)	1.12651 (16)	0.0387	
C212	1.32915 (16)	0.10684 (19)	1.04979 (17)	0.0364	
C213	1.25177 (15)	0.13052 (18)	0.97718 (15)	0.0302	
C214	0.67716 (14)	−0.10128 (16)	0.57445 (14)	0.0236	
N215	0.67192 (11)	−0.01687 (13)	0.52345 (11)	0.0219	
C216	0.59903 (14)	−0.00295 (17)	0.44840 (13)	0.0250	
C217	0.52933 (15)	−0.07246 (18)	0.42331 (15)	0.0301	
C218	0.53438 (15)	−0.15647 (18)	0.47575 (16)	0.0313	
C219	0.60967 (15)	−0.17207 (17)	0.55357 (15)	0.0283	
C220	0.59440 (16)	0.0891 (2)	0.39242 (16)	0.0360	
Ni1	0.782107 (17)	0.08387 (2)	0.593737 (17)	0.0203	
O301	1.09855 (15)	0.24209 (18)	0.67870 (18)	0.0708	
H1011	0.7847	0.3165	0.4569	0.0339*	
H1072	0.7179	0.4810	0.6438	0.0478*	
H1071	0.6229	0.4672	0.5673	0.0477*	
H1081	0.4917	0.5431	0.5955	0.0485*	
H1091	0.4416	0.6719	0.6645	0.0539*	
H1101	0.5415	0.7661	0.7840	0.0505*	
H1111	0.6912	0.7325	0.8329	0.0497*	
H1121	0.7424	0.6005	0.7663	0.0448*	
H1171	0.9264	−0.0389	0.3617	0.0437*	
H1181	0.9224	0.1205	0.2966	0.0455*	
H1191	0.8582	0.2559	0.3506	0.0391*	
H1203	0.8930	−0.1493	0.4602	0.0539*	
H1202	0.9043	−0.1012	0.5560	0.0535*	
H1201	0.8098	−0.1220	0.4829	0.0538*	
H2011	0.7692	−0.1714	0.6936	0.0297*	
H2071	1.0982	0.1653	0.8493	0.0443*	
H2072	1.0417	0.1611	0.9178	0.0448*	
H2091	1.1111	0.0447	1.0636	0.0436*	
H2101	1.2407	0.0027	1.1833	0.0494*	
H2111	1.3782	0.0415	1.1759	0.0471*	
H2121	1.3844	0.1256	1.0470	0.0452*	
H2131	1.2540	0.1624	0.9232	0.0378*	
H2171	0.4787	−0.0607	0.3691	0.0360*	
H2181	0.4875	−0.2023	0.4596	0.0384*	
H2191	0.6159	−0.2284	0.5925	0.0346*	
H2201	0.5398	0.0905	0.3400	0.0560*	
H2203	0.6441	0.0917	0.3726	0.0562*	
H2202	0.5977	0.1476	0.4299	0.0563*	
H3011	1.0477	0.2145	0.6805	0.1043*	
H3012	1.1023	0.3091	0.6914	0.1042*	

### Atomic displacement parameters (Å^2^)

**Table:**

	*U*^11^	*U*^22^	*U*^33^	*U*^12^	*U*^13^	*U*^23^
C101	0.0341 (12)	0.0252 (12)	0.0303 (11)	0.0032 (9)	0.0172 (9)	0.0072 (9)
N102	0.0270 (9)	0.0223 (9)	0.0239 (8)	0.0026 (7)	0.0113 (7)	0.0026 (7)
N103	0.0339 (10)	0.0215 (9)	0.0303 (9)	0.0042 (8)	0.0166 (8)	0.0015 (8)
C104	0.0235 (10)	0.0200 (10)	0.0236 (9)	0.0019 (8)	0.0078 (8)	−0.0018 (8)
S105	0.0394 (3)	0.0207 (3)	0.0304 (3)	0.0028 (2)	0.0197 (2)	−0.0005 (2)
S106	0.0285 (3)	0.0208 (3)	0.0249 (2)	0.0010 (2)	0.0132 (2)	0.0021 (2)
C107	0.0610 (17)	0.0215 (12)	0.0393 (13)	0.0018 (11)	0.0288 (12)	−0.0005 (10)
C108	0.0404 (14)	0.0302 (13)	0.0460 (14)	−0.0066 (11)	0.0136 (12)	0.0011 (11)
C109	0.0392 (15)	0.0336 (14)	0.0690 (18)	0.0103 (12)	0.0293 (14)	0.0146 (13)
C110	0.0639 (18)	0.0247 (13)	0.0444 (14)	0.0112 (12)	0.0321 (13)	0.0054 (11)
C111	0.0584 (17)	0.0292 (13)	0.0305 (12)	0.0087 (12)	0.0118 (12)	0.0007 (10)
C112	0.0400 (14)	0.0302 (13)	0.0339 (12)	0.0088 (11)	0.0108 (10)	0.0032 (10)
C113	0.0424 (14)	0.0195 (11)	0.0324 (11)	0.0063 (10)	0.0197 (10)	0.0059 (9)
C114	0.0241 (11)	0.0302 (12)	0.0239 (10)	0.0007 (9)	0.0092 (8)	0.0023 (9)
N115	0.0214 (9)	0.0290 (10)	0.0219 (8)	0.0015 (7)	0.0082 (7)	−0.0018 (7)
C116	0.0225 (11)	0.0325 (12)	0.0259 (10)	−0.0005 (9)	0.0075 (8)	−0.0042 (9)
C117	0.0338 (13)	0.0418 (14)	0.0364 (12)	0.0005 (11)	0.0200 (10)	−0.0087 (11)
C118	0.0348 (13)	0.0524 (16)	0.0320 (11)	−0.0011 (12)	0.0209 (10)	−0.0001 (11)
C119	0.0296 (12)	0.0406 (14)	0.0294 (11)	0.0029 (10)	0.0153 (9)	0.0083 (10)
C120	0.0416 (14)	0.0307 (13)	0.0392 (12)	0.0050 (11)	0.0224 (11)	−0.0038 (10)
C201	0.0290 (11)	0.0194 (10)	0.0249 (10)	−0.0003 (9)	0.0094 (9)	0.0011 (8)
N202	0.0222 (9)	0.0242 (9)	0.0211 (8)	0.0014 (7)	0.0070 (7)	−0.0004 (7)
N203	0.0241 (9)	0.0275 (10)	0.0227 (8)	0.0016 (8)	0.0037 (7)	0.0023 (7)
C204	0.0235 (11)	0.0281 (12)	0.0220 (9)	0.0026 (9)	0.0075 (8)	−0.0006 (9)
S205	0.0252 (3)	0.0250 (3)	0.0281 (3)	−0.0023 (2)	0.0067 (2)	0.0013 (2)
S206	0.0284 (3)	0.0307 (3)	0.0311 (3)	0.0018 (2)	−0.0007 (2)	−0.0003 (2)
C207	0.0326 (13)	0.0326 (13)	0.0344 (12)	−0.0023 (10)	0.0040 (10)	0.0010 (10)
C208	0.0299 (12)	0.0261 (12)	0.0254 (10)	−0.0003 (9)	0.0071 (9)	−0.0037 (9)
C209	0.0369 (13)	0.0387 (14)	0.0304 (11)	−0.0020 (11)	0.0143 (10)	−0.0012 (10)
C210	0.0555 (16)	0.0380 (14)	0.0221 (10)	0.0054 (12)	0.0121 (11)	0.0016 (10)
C211	0.0409 (14)	0.0349 (14)	0.0288 (11)	0.0107 (11)	−0.0010 (10)	−0.0073 (10)
C212	0.0296 (13)	0.0344 (14)	0.0427 (13)	−0.0014 (10)	0.0101 (10)	−0.0125 (11)
C213	0.0335 (12)	0.0288 (12)	0.0275 (10)	−0.0028 (10)	0.0102 (9)	−0.0037 (9)
C214	0.0265 (11)	0.0218 (11)	0.0238 (9)	0.0007 (9)	0.0109 (8)	−0.0019 (8)
N215	0.0233 (9)	0.0230 (9)	0.0208 (8)	0.0007 (7)	0.0098 (7)	−0.0018 (7)
C216	0.0233 (11)	0.0321 (12)	0.0210 (9)	0.0033 (9)	0.0096 (8)	−0.0020 (9)
C217	0.0235 (11)	0.0385 (13)	0.0251 (10)	0.0017 (10)	0.0052 (9)	−0.0061 (10)
C218	0.0246 (11)	0.0323 (13)	0.0362 (12)	−0.0043 (10)	0.0103 (9)	−0.0115 (10)
C219	0.0306 (12)	0.0227 (11)	0.0327 (11)	−0.0018 (9)	0.0127 (9)	−0.0026 (9)
C220	0.0284 (12)	0.0481 (15)	0.0274 (11)	0.0009 (11)	0.0053 (9)	0.0108 (11)
Ni1	0.02185 (14)	0.01956 (14)	0.02001 (12)	0.00104 (11)	0.00830 (10)	0.00119 (10)
O301	0.0684 (15)	0.0540 (14)	0.1051 (19)	−0.0185 (12)	0.0496 (14)	−0.0284 (13)

### Geometric parameters (Å, º)

**Table:**

C101—N102	1.286 (3)	C201—C214	1.454 (3)
C101—C114	1.453 (3)	C201—Ni1	2.854 (2)
C101—Ni1	2.854 (2)	C201—H2011	0.958
C101—H1011	0.949	N202—N203	1.379 (2)
N102—N103	1.386 (2)	N202—Ni1	2.0197 (18)
N102—Ni1	2.0127 (18)	N203—C204	1.312 (3)
N103—C104	1.305 (3)	C204—S205	1.708 (2)
C104—S105	1.754 (2)	C204—S206	1.764 (2)
C104—S106	1.714 (2)	S205—Ni1	2.4211 (6)
S105—C107	1.821 (2)	S206—C207	1.822 (3)
S106—Ni1	2.4259 (5)	C207—C208	1.514 (3)
C107—C113	1.507 (3)	C207—H2071	1.006
C107—H1072	0.970	C207—H2072	0.992
C107—H1071	0.958	C208—C209	1.384 (3)
C108—C109	1.395 (4)	C208—C213	1.382 (3)
C108—C113	1.383 (3)	C209—C210	1.379 (3)
C108—H1081	0.941	C209—H2091	0.954
C109—C110	1.375 (4)	C210—C211	1.379 (4)
C109—H1091	0.935	C210—H2101	0.936
C110—C111	1.371 (4)	C211—C212	1.384 (4)
C110—H1101	0.949	C211—H2111	0.947
C111—C112	1.385 (3)	C212—C213	1.385 (3)
C111—H1111	0.960	C212—H2121	0.946
C112—C113	1.384 (3)	C213—H2131	0.954
C112—H1121	0.956	C214—N215	1.357 (3)
C114—N115	1.358 (3)	C214—C219	1.387 (3)
C114—C119	1.385 (3)	N215—C216	1.338 (3)
N115—C116	1.344 (3)	N215—Ni1	2.1784 (17)
N115—Ni1	2.1787 (16)	C216—C217	1.399 (3)
C116—C117	1.399 (3)	C216—C220	1.485 (3)
C116—C120	1.490 (3)	C217—C218	1.366 (3)
C117—C118	1.371 (4)	C217—H2171	0.952
C117—H1171	0.937	C218—C219	1.387 (3)
C118—C119	1.386 (3)	C218—H2181	0.933
C118—H1181	0.947	C219—H2191	0.944
C119—H1191	0.946	C220—H2201	0.961
C120—H1203	0.964	C220—H2203	0.963
C120—H1202	0.959	C220—H2202	0.961
C120—H1201	0.962	O301—H3011	0.913
C201—N202	1.281 (3)	O301—H3012	0.907
			
N102—C101—C114	117.0 (2)	S206—C207—H2071	109.6
C114—C101—Ni1	78.89 (12)	C208—C207—H2071	112.0
N102—C101—H1011	121.8	S206—C207—H2072	108.5
C114—C101—H1011	121.1	C208—C207—H2072	110.9
Ni1—C101—H1011	159.4	H2071—C207—H2072	110.5
C101—N102—N103	116.68 (18)	C207—C208—C209	120.7 (2)
C101—N102—Ni1	118.13 (15)	C207—C208—C213	120.1 (2)
N103—N102—Ni1	124.68 (13)	C209—C208—C213	119.2 (2)
N102—N103—C104	111.49 (17)	C208—C209—C210	120.5 (2)
N103—C104—S105	116.56 (16)	C208—C209—H2091	120.0
N103—C104—S106	129.35 (16)	C210—C209—H2091	119.5
S105—C104—S106	114.09 (11)	C209—C210—C211	120.2 (2)
C104—S105—C107	101.58 (10)	C209—C210—H2101	119.6
C104—S106—Ni1	93.07 (7)	C211—C210—H2101	120.2
S105—C107—C113	108.07 (15)	C210—C211—C212	119.7 (2)
S105—C107—H1072	109.2	C210—C211—H2111	120.6
C113—C107—H1072	110.0	C212—C211—H2111	119.7
S105—C107—H1071	109.0	C211—C212—C213	119.9 (2)
C113—C107—H1071	110.8	C211—C212—H2121	120.4
H1072—C107—H1071	109.7	C213—C212—H2121	119.6
C109—C108—C113	120.3 (2)	C212—C213—C208	120.5 (2)
C109—C108—H1081	119.7	C212—C213—H2131	120.5
C113—C108—H1081	120.0	C208—C213—H2131	119.1
C108—C109—C110	120.0 (3)	C201—C214—N215	115.87 (18)
C108—C109—H1091	119.2	C201—C214—C219	121.1 (2)
C110—C109—H1091	120.8	N215—C214—C219	122.99 (19)
C109—C110—C111	120.1 (2)	C214—N215—C216	118.19 (18)
C109—C110—H1101	120.5	C214—N215—Ni1	110.22 (13)
C111—C110—H1101	119.4	C216—N215—Ni1	131.00 (15)
C110—C111—C112	119.9 (2)	N215—C216—C217	121.3 (2)
C110—C111—H1111	119.4	N215—C216—C220	117.78 (19)
C112—C111—H1111	120.7	C217—C216—C220	120.91 (19)
C111—C112—C113	120.9 (2)	C216—C217—C218	120.2 (2)
C111—C112—H1121	118.7	C216—C217—H2171	119.2
C113—C112—H1121	120.3	C218—C217—H2171	120.6
C107—C113—C112	120.1 (2)	C217—C218—C219	119.1 (2)
C107—C113—C108	121.2 (2)	C217—C218—H2181	120.2
C112—C113—C108	118.7 (2)	C219—C218—H2181	120.6
C101—C114—N115	115.90 (18)	C218—C219—C214	118.2 (2)
C101—C114—C119	120.8 (2)	C218—C219—H2191	122.2
N115—C114—C119	123.3 (2)	C214—C219—H2191	119.6
C114—N115—C116	118.50 (18)	C216—C220—H2201	110.4
C114—N115—Ni1	110.48 (13)	C216—C220—H2203	110.4
C116—N115—Ni1	130.96 (15)	H2201—C220—H2203	110.2
N115—C116—C117	120.6 (2)	C216—C220—H2202	108.9
N115—C116—C120	118.15 (19)	H2201—C220—H2202	109.3
C117—C116—C120	121.3 (2)	H2203—C220—H2202	107.4
C116—C117—C118	120.5 (2)	N115—Ni1—N215	93.28 (6)
C116—C117—H1171	117.9	N115—Ni1—S106	158.09 (5)
C118—C117—H1171	121.6	N215—Ni1—S106	89.15 (4)
C117—C118—C119	119.3 (2)	N115—Ni1—S205	92.82 (5)
C117—C118—H1181	119.8	N215—Ni1—S205	158.24 (5)
C119—C118—H1181	121.0	S106—Ni1—S205	92.92 (2)
C118—C119—C114	117.9 (2)	N115—Ni1—N202	108.62 (7)
C118—C119—H1191	122.3	N215—Ni1—N202	77.43 (7)
C114—C119—H1191	119.9	S106—Ni1—N202	93.17 (5)
C116—C120—H1203	109.1	S205—Ni1—N202	80.83 (5)
C116—C120—H1202	110.4	N115—Ni1—N102	77.72 (7)
H1203—C120—H1202	109.7	N215—Ni1—N102	110.44 (7)
C116—C120—H1201	110.4	S106—Ni1—N102	81.03 (5)
H1203—C120—H1201	108.1	S205—Ni1—N102	91.27 (5)
H1202—C120—H1201	109.1	N202—Ni1—N102	169.99 (7)
N202—C201—C214	116.97 (19)	N115—Ni1—C201	111.11 (6)
C214—C201—Ni1	78.68 (12)	N215—Ni1—C201	54.55 (6)
N202—C201—H2011	120.8	S106—Ni1—C201	87.95 (4)
C214—C201—H2011	122.2	S205—Ni1—C201	103.85 (5)
Ni1—C201—H2011	158.4	N202—Ni1—C201	23.38 (6)
C201—N202—N203	116.94 (18)	N115—Ni1—C101	54.55 (6)
C201—N202—Ni1	117.90 (14)	N215—Ni1—C101	111.35 (6)
N203—N202—Ni1	124.35 (14)	S106—Ni1—C101	104.43 (5)
N202—N203—C204	112.84 (17)	S205—Ni1—C101	89.11 (5)
N203—C204—S205	127.77 (16)	N202—Ni1—C101	160.18 (6)
N203—C204—S206	109.85 (16)	N102—Ni1—C201	161.75 (7)
S205—C204—S206	122.38 (13)	N102—Ni1—C101	23.42 (6)
C204—S205—Ni1	94.18 (7)	C201—Ni1—C101	161.74 (6)
C204—S206—C207	104.73 (11)	H3011—O301—H3012	112.0
S206—C207—C208	105.08 (16)		

### Hydrogen-bond geometry (Å, º)

**Table:**

*D*—H···*A*	*D*—H	H···*A*	*D*···*A*	*D*—H···*A*
O301—H3011···S205	0.91	2.42	3.323 (3)	173
O301—H3012···N203^i^	0.91	2.04	2.919 (4)	162

Symmetry code: (i) −*x*+2, *y*+1/2, −*z*+3/2.
